# Silicon and Silicon Carbide Recrystallization by Laser Annealing: A Review

**DOI:** 10.3390/ma16247674

**Published:** 2023-12-16

**Authors:** Daniele Arduino, Stefano Stassi, Chiara Spano, Luciano Scaltrito, Sergio Ferrero, Valentina Bertana

**Affiliations:** 1 Department of Applied Science and Technology, Politecnico di Torino, Corso Duca degli Abruzzi 24, 10129 Torino, Italy; daniele_arduino@polito.it (D.A.); chiara.spano@polito.it (C.S.); luciano.scaltrito@polito.it (L.S.); sergio.ferrero@polito.it (S.F.); valentina.bertana@polito.it (V.B.); 2 Department of Information Engineering, Electrical Engineering and Applied Mathematics, University of Salerno, Via Giovanni Paolo II 132, 84084 Fisciano, Italy

**Keywords:** laser annealing, silicon, silicon carbide, crystallization, integrated devices

## Abstract

Modifying material properties within a specific spatial region is a pivotal stage in the fabrication of microelectronic devices. Laser annealing emerges as a compelling technology, offering precise control over the crystalline structure of semiconductor materials and facilitating the activation of doping ions in localized regions. This obviates the necessity for annealing the entire wafer or device. The objective of this review is to comprehensively investigate laser annealing processes specifically targeting the crystallization of amorphous silicon (Si) and silicon carbide (SiC) samples. Silicon finds extensive use in diverse applications, including microelectronics and solar cells, while SiC serves as a crucial material for developing components designed to operate in challenging environments or high-power integrated devices. The review commences with an exploration of the underlying theory and fundamentals of laser annealing techniques. It then delves into an analysis of the most pertinent studies focused on the crystallization of these two semiconductor materials.

## 1. Introduction

The rapid diffusion of microelectronics and their advancement toward improved performance and reduced size has necessitated the development of new technological processes for selectively modifying material properties in specific regions without affecting others. For instance, there is a need to locally crystallize amorphous materials to achieve new properties or activate dopant ions that have been previously implanted in a semiconductor region. This can be accomplished through semiconductor annealing. Traditional annealing methods involve heating samples to high temperatures (above 1000 °C) using furnaces to repair lattice damage and electrically activate dopants.

Thermal annealing is a critical step in semiconductor device fabrication that is typically performed after other processes. Subjecting the entire sample to high temperatures can have undesirable side effects on device functionality, including impurity redistribution and damage to implanted ion disposition [1]. Moreover, it is incompatible with prior metal deposition, as the elevated temperature can cause metal connections to melt and redistribute.

In recent years, various alternatives to thermal annealing in furnaces have been explored, with laser annealing emerging as one of the most effective technologies [2]. Depending on the light frequency, laser annealing offers the advantage of intense absorption on a thin surface layer (a few nanometers deep). This capability allows for the generation of the extremely high temperatures necessary for annealing lattice damage or crystallizing amorphous films in a precisely localized region. Importantly, this occurs without affecting the rest of the sample, mitigating the risk of unwanted alterations to the device [3].

During the laser annealing process, the material phase (crystallinity), optical properties (refractive index, light absorption), and electrical properties (conductivity, energy bandgap, direct or indirect bandgap) of the samples are altered through light absorption [4]. The incident laser energy is absorbed through electronic excitations and rapidly transferred to the lattice, resulting in the melting of the crystal to a certain depth and inducing lattice damage. Subsequently, liquid phase epitaxial regrowth occurs from the undamaged substrate underneath, leading to recrystallization of the melted region into nearly perfect single-crystal material, with dopants occupying substitutional sites in the lattice, thereby activating the implanted ions [5].

Laser annealing is a versatile technique applicable to various types of semiconductors, including group IV semiconductors like silicon, germanium [6], and silicon carbide, as well as III-V compound semiconductors like gallium arsenide [7,8].

**Figure 1:**
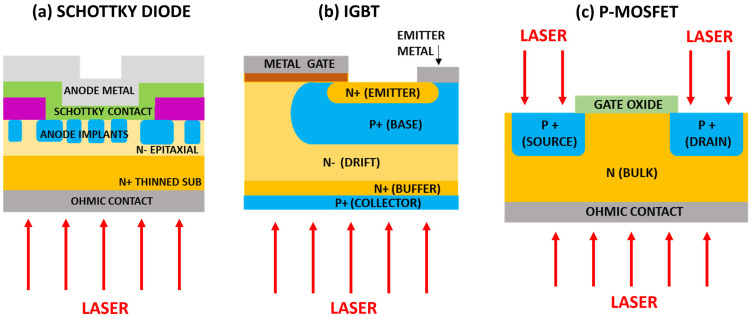
Scheme of the laser annealing process exploited to (**a**) form ohmic contact on the Schottky diode, (**b**) activate doping on the collector layer of IGBT, and (**c**) activate doping in the source and drain region and form ohmic contact in a MOSFET (in this case a pMOSFET, although the process is the same for nMOSFET).

Laser thermal annealing is an ultrafast and low thermal budget process solution for the passivation of backside illuminated sensors and power devices. Laser annealing can be a solution for the backside contact of those chips with a vertical flow of electrical current, where an ohmic contact and/or collector on the wafer backside are required [9,10], including SiC power Metal-Oxide-Semiconductor Field Effect Transistors (MOSFET) [11], Insulated Gate Bipolar Transistor (IGBT) [12], and high voltage diodes [13,14]. Moreover, this process can also be useful for the ohmic contact formation process in SiC Schottky diodes, causing a negligible impact on the device’s front side [15,16]. The laser annealing process has also been used to activate doping ions in the source and drain region of MOSFET to avoid damage to the channel region induced by global device heating processes such as rapid thermal annealing (RTA) [17,18,19]. A scheme of laser annealing processes in semiconductor devices is reported in Figure 1. The high-temperature annealing region is restricted to thin layers while keeping underlying layers at low temperatures. An ultrafast annealing time and proper laser parameters may achieve high performance and high yields, locking in the surface properties without damaging buried device layers [20].

This study aims to provide a comprehensive review of laser annealing processes specifically focused on the crystallization of amorphous silicon (Si) and silicon carbide (SiC) samples. Silicon is widely used in microelectronics and solar cell applications, while SiC is vital for the development of high-power integrated components and devices operating in harsh environments and at high temperatures [21,22]. SiC has also garnered attention for optical applications due to its nonlinear optical properties [23]. The review includes a brief overview of the underlying theory and principles of laser annealing, followed by a presentation of the most relevant studies that focus on the crystallization of the amorphous layer of these two semiconductor materials. This study provides a critical analysis of various processes reported in the literature. The studies are categorized based on the laser wavelength employed, and a detailed examination of the process parameters of the systems involved is conducted. Additionally, the main crystallization results obtained are outlined, accompanied by a discussion of the outcomes of the primary characterization methods employed in these studies.

## 2. Laser Annealing Theory

Laser annealing is a technique that involves using light absorption to deliver energy to a material. Typically, laser annealing is limited to thin surface layers because the intensity of the incident light decreases as it penetrates the material, based on the material’s absorption coefficient, *α*. The Beer–Lambert law describes this decay of intensity with depth *z* using the equation:

(1) $$I(z)=I_{0}e^{-\alpha z}$$

where *I*_0_ is the intensity on the surface.

The optical penetration or absorption depth, defined as the depth at which the transmitted light’s intensity drops to 1/*e* (about 37%) of its initial value on the surface, is denoted as *δ =* 1/*α*. Both *α* and *δ* depend on the semiconductor type, laser wavelength, and temperature.

Since energy absorption is primarily confined to the absorption depth, this parameter is applicable to all beam profiles, even though it was originally developed for plane waves. Consequently, it is possible to locally modify surface properties without altering the bulk material by using laser wavelengths with short absorption depths [24].

In continuous wave (CW) or nanosecond laser pulses, it is generally assumed that single-photon interactions account for most of the absorption. However, in the case of picosecond (ps) and femtosecond (fs) lasers, the absorption depths can be reduced due to phenomena such as optical breakdown and multiphoton absorption, which result from the extremely high instantaneous intensity of these lasers [25].

**Figure 2:**
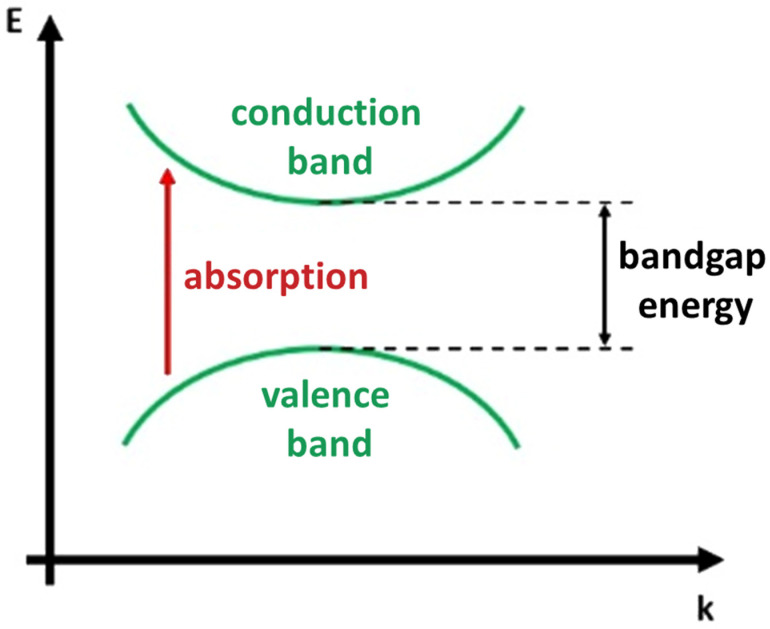
Scheme of the material interband photon absorption.

The absorption of laser light in insulators and semiconductors is typically achieved through resonant excitations, such as transitions of valence band electrons to the conduction band (interband transitions, Figure 2) or within bands (intersubband transitions) [26].

Typically, photons interact with the electronic or vibrational states present in a material based on their energy. Laser photons of energy greater than that of the band gap generate electron-hole pairs promoted to states of higher kinetic energy in the conduction and valence band. These excited electronic states can subsequently transfer energy to lattice phonons, becoming heat. If there are no other factors such as impurity or defect states or multiphoton absorption, photons with energy lower than the material’s band gap will not be absorbed. This generally corresponds to light wavelengths within the infrared to visible spectrum for semiconductors and within the vacuum ultraviolet range (below 200 nm) for insulators.

The laser field to the electronic system is quickly transferred to phonons in less than 1 picosecond, leading to the melting of the near-surface region [27]. The near-surface region of a sample can melt and stay molten for a thermalization time depending on the material treated, during which dopant diffusion in the liquid state and nonequilibrium segregation occur together with ultrarapid recrystallization [28]. The crystallization process can be described in terms of models based on macroscopic diffusion equations for heat and mass transport. The mechanism can be explained in terms of a molten layer that extends all over the amorphous thickness and whose subsequent solidification occurs on a crystalline seed, like a liquid phase epitaxy [27]. In this way, the laser treatment allows the annealing and treatment of the lattice damage caused by ion implantation, diffuse surface-deposited dopant films, and recrystallized doped amorphous films deposited on the substrate [28].

The specific material and its mechanisms determine the time required for excited electronic states to transfer energy to phonons and thermalize. For non-metals, the thermalization time can be from 10^−8^ s to 10^−6^ s, while it is around 10^−12^–10^−10^ s for metals [25]. If the laser-induced excitation rate is lower than the thermalization rate, the transient electronically excited states are not significant and the absorbed laser energy can be considered directly converted into heat. This process is known as photothermal (pyrolytic) processing and is commonly observed in semiconductor laser processing with long pulse times (>ns). During this process, material response can be analyzed purely in thermal terms [24].

However, when the laser-induced excitation rate exceeds the thermalization rate, significant excitations can accumulate in the intermediate states. This can lead to direct bond-breaking due to the excitation energies, resulting in non-thermal material modifications. This phenomenon is referred to as photochemical (photolytic) processing, where there are no changes in the system’s temperature. Ultrafast femtosecond laser pulses with short-wavelength light, where the photon energy is comparable to the chemical bond energy, can trigger photochemical processing [29].

The laser annealing process is significantly influenced by the technology of the implemented system, which determines the wavelength and the shortest pulse duration of the laser. Several key parameters of the laser system can be adjusted to control the effects of the annealing process on the material surface. A list of the most crucial system specifications and parameters involved in laser annealing processes is provided below. These descriptions aim to enhance understanding of the main results in silicon and silicon carbide annealing processes described in the following sections.

Laser technology: The laser relies on a certain physical mechanism depending on the laser material, which affects the other laser parameters (such as its power, pulse duration, etc.). Possible laser technologies range from gas lasers (such as CO_2_ laser) to excimer lasers (based on a combination of a noble gas and a reactive gas, such as KrF and XeCl lasers) and solid-state lasers (based on doped crystal, such as Nd:YAG lasers).

Wavelength: This parameter plays a significant role in the annealing process as it affects light absorption efficiency and the depth of laser effects in the material. Generally, the penetration depth is proportional to *λ*/4*πk* with *k*, which is the absorption coefficient. The light wavelength (which is directly related to photon energy by the Planck equation) must fit with the material bandgap to be absorbed, otherwise, photons pass through the material. Laser wavelengths used for annealing range from infrared to visible and ultraviolet (UV) regions.

Power and spot size: A combination of these two parameters defines the power density, which indicates the energy quantity delivered by the laser beam to the desired target per unit of area and time. The energy density or fluence represents the energy transferred per unit area by a single pulse. The use of a magnifying lens can increase the given number of photons directed to a specific area, raising the laser fluence and thus the target temperature faster.

Pulse duration. This is the time between the beginning and end edges of an energy pulse, often measured at full-width half maximum (FWHM). Lasers treated in this review are characterized by nanosecond or femtosecond pulses. Generally, considering a fixed frequency, short pulses allow samples to cool down between bursts of light and, hence protect illuminated samples from overheating. However, in general, short pulses with high peak powers (as in the case of femtosecond lasers) may ablate the surface material, avoiding heating the surrounding area. Obviously, this parameter is absent in the case of continuous wave lasers.

Scanning speed: This parameter represents the velocity of the relative motion between the laser and the material. Slower scanning speeds result in the laser remaining in a particular spatial position on the material for a longer time, leading to higher annealing effects.

Beam profile: The laser beam can have various profiles, such as Gaussian, multimode, or rectangular. The energy distribution of the beam determines the spatial effect of the annealing process, yielding different results. Gaussian beams, for example, cause stronger effects near the beam center and weaker effects near the edges, while rectangular beams provide a more uniform spatial distribution.

Environment: Since laser annealing increases the local temperature of the material, environmental conditions can impact process outcomes. The presence of air can cause reactions between the heated material and atmospheric gases, which is not observed in a vacuum or an argon (Ar) atmosphere.

Manipulating these main parameters is possible by taking advantage of the main components of the laser annealing equipment, which consists of a laser source composed of a laser head (which determines the emitted wavelength, frequency, and pulse duration), lenses, and filters for shaping the laser beam profile.

A laser stage motor is normally responsible for the scan speed. Indeed, the laser head can be based on the technology called plotter laser, where the laser light is driven by some fixed internal mirrors, and its movement on the sample surface is controlled by a stage motor that determines the scan speed and the resolution on the X and Y axes. An alternative technology is the galvo head, where the laser ray is driven by rotating dynamic internal mirrors, and a stage motor is used only for large displacement.

## 3. Silicon Laser Annealing

Silicon is a crucial material for creating microelectronic devices, and various fabrication processes have been developed to manipulate its properties based on specific applications. One important technique being studied is its transformation from amorphous silicon to polycrystalline silicon, which combines the advantages of both single-crystal silicon and amorphous silicon [30]. While amorphous silicon has lower carrier mobility, it is cost-effective to manufacture. On the other hand, polycrystalline silicon, though inferior in mechanical and electrical properties compared to single-crystal silicon, finds extensive use in the field of optoelectronics.

Currently, laser annealing is the most commonly employed technology for preparing polysilicon materials. This method involves the use of a high-power pulsed laser on an amorphous silicon sample. The surface of the amorphous silicon absorbs the laser energy, causing the temperature to rise to the phase transition point. After cooling and solidification, the amorphous silicon is transformed into polysilicon [31,32].

This process exhibits short annealing time and high crystallization efficiency. Additionally, laser annealing keeps the substrate at a low temperature, reducing the requirements on the substrate material and thus lowering manufacturing costs.

The crystallization of silicon grains is influenced by energy density, pulse duration, and laser shape; however, laser wavelength is the primary parameter governing the crystallization phenomenon due to its role in optical absorption [33].

Specifically, crystallization occurs when the photon energy (associated with the photon wavelength) matches the bandgap of the material. The light induces a transition between the ground and excited states, resulting in the loss of a photon and the production of an excited state [34]. In the case of silicon, which has a bandgap energy of 1.1 eV, the minimum wavelength required for electron transition from the valence to the conduction band is around 1100 nm, corresponding to the infrared (IR) region.

**Figure 3:**
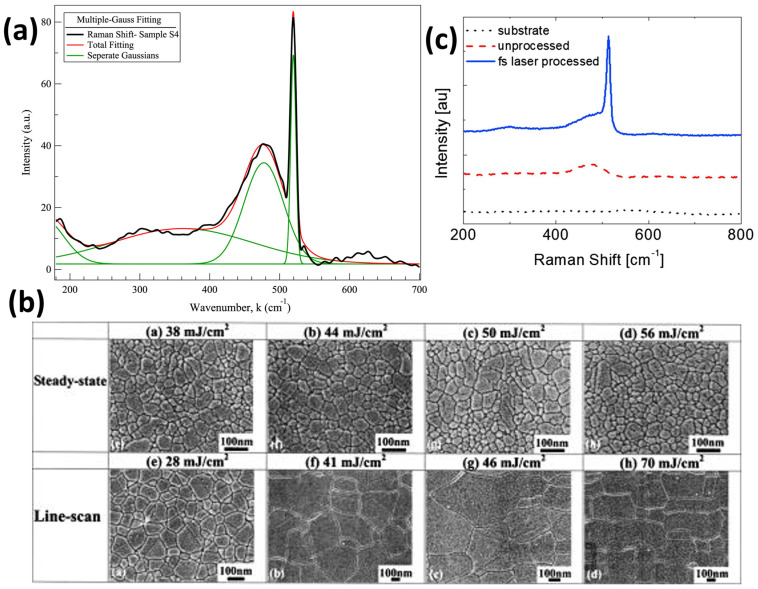
(**a**) Raman spectrum of annealed Silicon sample (black). The green curves are separated Gaussian fits for different scattering modes, while the red curve is the sum of the fits. Reprinted from [35], Copyright (2019), with permission from Elsevier. (**b**) SEM pictures of crystalline grains in Silicon samples after femtosecond laser annealing processes with different configurations and fluences. Reprinted from [36], with the permission of AIP Publishing. (**c**) Raman spectra of bare glass substrate and amorphous silicon film without (dash) and with (solid) femtosecond laser processing. Reprinted from [37], Copyright (2019), with permission from Elsevier.

Several experiments have been conducted on silicon laser annealing using IR laser sources. Salman et al. [35] successfully crystallized a silicon sample using an IR-pulsed laser with a wavelength of 1064 nm (photon energy of 1.165 eV) and a pulse duration of 200 ns. The experiment was performed under ambient conditions using a pulsed fiber laser built on Ytterbium-doped active fiber, with an average optical power of 20 W and a maximum pulse energy of 0.50 mJ. The repetition rate was set to 20 kHz. Successful crystallization was confirmed through Raman spectroscopy analysis, showing a main scattering peak at 520 cm^−1^, which is typical of a crystalline silicon structure (Figure 3a). The crystallization process was also verified using Fourier Transform Infrared (FTIR) spectroscopy, Scanning Electron Microscopy (SEM), Transmission Electron Microscopy (TEM), and Atomic Force Microscopy (AFM), which are powerful analysis tools for investigating semiconductor crystalline structures.

Previous studies [36,38] have utilized infrared laser radiation with a wavelength of 800 nm, generated by a Ti:Sapphire laser system with femtosecond pulse duration. Despite this difference, the presence of crystalline grains can be observed from AFM, SEM (Figure 3b), and TEM measurements, indicating the successful attainment of polysilicon through the annealing process.

Other groups [37,39] have demonstrated the effectiveness of IR radiation (λ = 800 nm by Zhan et al. [37] and λ = 1026 nm by Bronnikov et al. [39]) for silicon crystallization using laser systems based on different technologies (Yb:KGW laser system). Before the laser annealing process, the Raman spectra of the as-grown amorphous film display a broad peak, ranging from 420 to 530 cm^−1^, associated with the optical vibration modes of amorphous silicon [40,41]. However, after the annealing step, a distinct crystalline peak at 513 cm^−1^ becomes evident (Figure 3c). The sharp peak exhibits a blue shift of approximately 7 cm^−1^ compared to the peak of single crystalline silicon due to phonon confinement in nanocrystalline silicon and mechanical stress induced in the lattice by femtosecond laser treatment [40].

**Figure 4:**
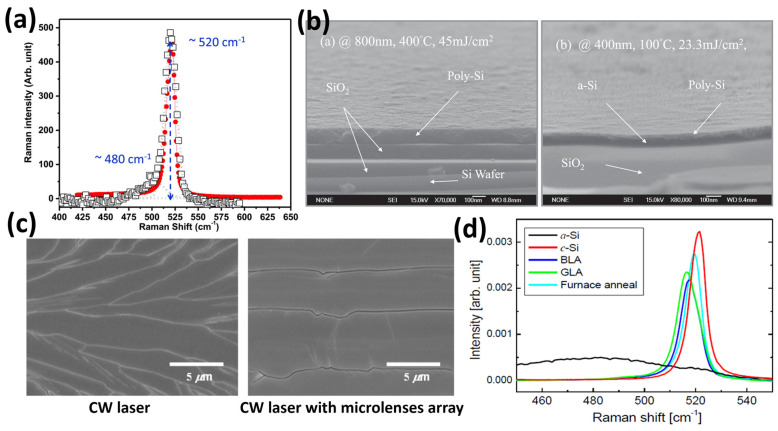
(**a**) Raman spectra of silicon dies showing the peak at 520 cm^-1^ (typical of crystalline Si) for the sample annealed using a 400 nm laser wavelength (sharper, red circles and red curve) and for the sample annealed using an 800 nm laser (smaller, black open squares) [45]. (**b**) Cross-sectional SEM images of samples annealed by femtosecond laser annealing with IR (800 nm, 400 °C, 45 mJ/cm^2^, left) and UV (400 nm, 100 °C, 23.3 mJ/cm^2^, right) radiation, showing the formation of poly-Si. The scale bars correspond to 100 nm [45]. (**c**) SEM images of crystallized poly-Si film treated with (left) conventional CW laser and (right) CW laser equipped with a cylindrical microlens array. The scale bars correspond to 5 µm. Copyright 2012, Wiley. Reproduced with permission from Ref. [53]. (**d**) Raman spectra of a-Si, c-Si, and polysilicon samples treated with BLA, GLA, and furnace annealing [54].

Laser annealing experiments have also explored more energetic wavelengths in the near UV range (200–400 nm) [42] and visible range (400–780 nm) [43,44]. For instance, Pan et al. utilized near ultraviolet (λ ≈ 400 nm) femtosecond laser annealing in a scanning mode to crystallize amorphous silicon (a-Si) films at room temperature. They investigated the impact of laser fluence, beam overlap, and number of laser shots on the average grain size of the resulting polycrystalline silicon. The experiment revealed that increasing either the beam overlap at a fixed fluence or the fluence for a fixed number of shots generally leads to larger grain sizes [45]. Additionally, they compared the crystallization degree of the polysilicon obtained through UV annealing with that produced by IR annealing (λ ≈ 800 nm) using Raman spectroscopy. Figure 4a demonstrates that the Raman peak associated with λ = 400 nm is sharper than the peak associated with λ = 800 nm, indicating a higher crystallization degree at λ = 400 nm (near UV) compared to λ = 800 nm (near-infrared).

In contrast, cross-sectional SEM images showed that the 100 nm thick a-Si film is not fully crystallized by UV annealing, unlike in the case of IR annealing (see Figure 4b). This can be attributed to the much shorter penetration depth of 400 nm light in amorphous silicon compared to 800 nm light.

UV radiation with nanosecond pulse durations has also been tested [46,47,48,49], as demonstrated by Garcia et al. [33]. They employed solid-state laser systems for annealing and compared the effect of UV radiation with that of visible light. The Nd:YVO_4_ systems, with pulse widths of 15 ns and 12 ns at a repetition rate of 50 kHz, were used at different wavelengths: fundamental frequency in the IR (1064 nm), doubled to green (532 nm), and tripled to UV (355 nm). Two fluence thresholds (F1 and F2) governing the melting process were identified, with their values depending on the wavelength employed. These thresholds represent the range of fluence within which the annealing process should take place. When the fluence is lower than F1, the silicon surface is not melted as the energy of the laser pulse is insufficient for a phase change. On the other hand, exceeding the F2 threshold results in ablation and material damage. For a UV wavelength of 355 nm, the F1 and F2 values are 70 mJ/cm^2^ and 374 mJ/cm^2^, respectively, while for a visible wavelength of 532 nm, F1 and F2 values are 110 mJ/cm^2^ and 304 mJ/cm^2^, respectively [33]. The authors were not able to crystallize a-Si with their system using a 1064 nm wavelength, because they had to use a longer laser pulse and higher fluence due to lower absorption in the IR range, causing material ejection.

Finally, laser annealing and crystallization of amorphous Si have also been explored using visible light generated by nanosecond pulsed and continuous wave lasers [50,51,52]. Son et al. [53] employed a Nd:YVO_4_ continuous wave (CW) laser with a wavelength of 532 nm (green light), an output power of 7.5 W, and a scanning speed of 270 mm/s to crystallize the samples. The laser beam had dimensions of 20 μm (short axis, scan direction) × 800 μm (long axis, transverse direction), with a Gaussian shape in the short axis and a top-hat shape in the long axis. This conventional laser system was compared with a system that utilized a cylindrical microlens array. In the latter system, the laser beam was split into two components: one traveled directly while the other was refracted through the cylindrical microlens. The two components met and interference occurred due to the superposition of the split beams, resulting in enhanced intensity and incident power per unit area on the a-Si due to constructive interference.

SEM analysis (Figure 4c) depicted the poly-Si film annealed using the conventional CW laser and the CW laser equipped with a microlens array. The grains of the poly-Si film produced by conventional CW laser crystallization were large but had irregular shapes and orientations. In contrast, the grains obtained by CW laser crystallization using a cylindrical microlens array were larger and more regular.

In order to provide a comprehensive analysis of annealing in the visible range, Pyo et al. [54] investigated the polycrystalline silicon obtained through green laser annealing (GLA) using a 532 nm nanosecond pulsed laser and blue laser annealing (BLA) using a 450 nm continuous wave laser. For GLA, the second harmonic of a Q-switched Nd:YAG laser was utilized with a scanning speed of 4 cm/s, a pulse repetition rate of 30 kHz, and a pulse width of 30 ns. The beam profile was approximately Gaussian with a beam waist of approximately 6 μm, and the peak power was 2.7 W, corresponding to a pulse energy of 86 nJ and an energy density of 153 mJ/cm^2^. The CW laser used for BLA consisted of two GaN-based blue laser diodes emitting at 450 nm. The BLA beam shape was rectangular, with a spot size of 6 μm × 2 μm and a power of 530 mW. The laser annealing experiments were conducted at room temperature (25 °C) under a nitrogen environment, with nitrogen pressure maintained at 1.5 times the atmospheric pressure.

After the annealing processes, the crystallinity of the annealed poly-Si was examined using Raman spectroscopy, as shown in Figure 4d, and compared with a standard furnace heating process. The Raman spectrum of amorphous Si exhibited a broad distribution centered at approximately 480 cm^−1^ in the transverse optic (TO) phonon mode, while crystalline Si displayed a sharp peak at around 520 cm^−1^ associated with the TO phonons. The shift and full width at half maximum (FWHM) of the a-Si and c-Si peaks were used to analyze the crystallinity of the annealed poly-Si film. Improved crystallinity is indicated by a shift in the TO phonon peak of poly-Si towards that of c-Si. The Raman shift values for furnace annealing, BLA, and GLA were 519 cm^−1^, 517.5 cm^−1^, and 516.5 cm^−1^, respectively. The FWHM values for furnace annealing, BLA, and GLA were 7.5 cm^−1^, 9.0 cm^−1^, and 9.5 cm^−1^, respectively.

By using the relative values of FWHM and the intensity of TO phonon Raman peaks, it is possible to calculate the crystal volume fraction (*f_c_*) of poly-Si [55]. The *f_c_* of poly-Si can be quantitatively evaluated using the following formula:

(2) $$f_{c}=\frac{I_{c}}{I_{c}+I_{a}}$$

where *f_c_* is the crystalline volume fraction. *I_c_* and *I_a_* stand for the integrated Raman scattering intensity of crystalline and amorphous sections, respectively. *I_c_* can be obtained by the deconvolution of each Raman spectrum into Gaussian components corresponding to the crystalline phases. The same can be done for *I_a_*, but with the amorphous phases. Looking at Figure 4d, when a-Si becomes poly-Si, the TO phonon peak of the crystalline phase is slowly shifted toward the c-Si peak and FWHM is decreased, resulting in an increase in *f_c_*. The fc values were 90.6% for BLA and 88.2% for GLA, indicating a slightly higher crystal quality for BLA. This finding is consistent with the longer Raman peak shift of GLA observed in Figure 4d. As expected, the furnace-annealed poly-Si sample showed the best crystallinity, while BLA and GLA showed lower crystallinity conversion. On the other hand, BLA and GLA processes have the advantage of heating only the superficial part of the sample, with respect to furnace processes, obtaining a successful degree of crystalline conversion.

A summary of the main parameters used during Si laser annealing processes described in the literature, which successfully convert amorphous silicon into crystalline or polycrystalline silicon, is presented in Table 1.

Research has revealed the potential for transforming amorphous silicon (a-Si) into poly-Si using a range of wavelengths spanning from infrared (IR) to ultraviolet (UV) across different laser systems. Silicon exhibits reduced absorption of IR light compared to visible and UV light, necessitating the use of higher power and shorter pulse durations in these procedures. Despite the challenges associated with crystallization using IR light, it is feasible even on thicker amorphous layers due to its greater penetration depth; conversely, laser annealing processes facilitate crystallization more readily with visible and UV lasers, proving more effective on thin amorphous layers due to the higher absorption rate of silicon.

## 4. Silicon Carbide Laser Annealing

Silicon carbide (SiC) is a valuable alternative to silicon for applications that involve high temperatures and harsh, corrosive environments that would damage standard silicon and polymer electronics [56,57,58,59,60,61]. The literature includes various examples of high-temperature pressure sensors [62,63,64,65,66,67,68], accelerometers [69], and micromotors [70,71] fabricated using SiC.

Furthermore, the bandgap of both amorphous and crystalline forms of silicon carbide (SiC), which is wider than the Si bandgap, makes it a promising material for optoelectronic applications [72,73], as well as high-power and RF/microwave electronics [74,75,76,77].

The crystalline form of SiC (c-SiC) is particularly suitable for high-power applications due to its large bandgap (3–3.3 eV), high thermal conductivity (4.9 W cm^−1^ K^−1^), high breakdown electric field strength (2.2 × 10^6^ V cm^−1^), and high saturated electron drift velocity (2.0 × 10^7^ cm s^−1^) [72].

Different arrangements of Si-C bilayers in the [0001] direction give rise to over 200 polytypes of SiC crystal structures [78]. These polytypes, named with the designations C, H, or R to represent cubic, hexagonal, or rhombohedral symmetry, exhibit significant variations in properties such as carrier mobility and electronic gap. Among them, 4H-SiC is often favored for microelectronics applications due to its high carrier mobility.

Lasers have been investigated as tools for both additive (annealing, deposition, surface alteration, and doping) and subtractive (ablation) SiC processing since the early 1980s; however, they have not been widely adopted for microelectronics and MEMS applications. Various types of lasers have been tested for microfabrication on silicon carbide, including traditional excimer, Nd:yttrium aluminum (Nd:YAG), and CO_2_ lasers [79,80,81,82,83,84], as well as more recent lasers such as N_2_ [85], Ar^+^ [86], Cu vapor [87], and promising picoseconds [88] and femtosecond lasers [89,90].

For MEMS applications, annealing of amorphous and polycrystalline SiC can be useful for surface recovery after ion implantation damage [75] or to transform amorphous silicon carbide into a crystalline phase [91,92].

A subject of ongoing discussion is whether laser-induced recrystallization occurs through solid-state [93,94,95] or liquid-phase recrystallization [93,94,96,97,98,99,100]. Different effects have been reported by different research groups using the same type of laser. For example, Hishida et al. [95] reported solid-phase recrystallization while Ahmed et al. [99] reported liquid-phase recrystallization even though both groups used XeCl lasers to anneal Al^+^ ion-implanted 6H-SiC.

In general, various types of lasers, capable of generating pulsed or continuous wave radiation at different wavelengths, can be used for SiC laser annealing. However, nanosecond-pulsed UV lasers such as excimer and frequency tripled and quadrupled Nd:YAG lasers (base emission at 1064 nm) are the most commonly used due to their prevalence and the high optical absorption of crystalline SiC at UV wavelengths [91,92,101,102,103].

**Figure 5:**
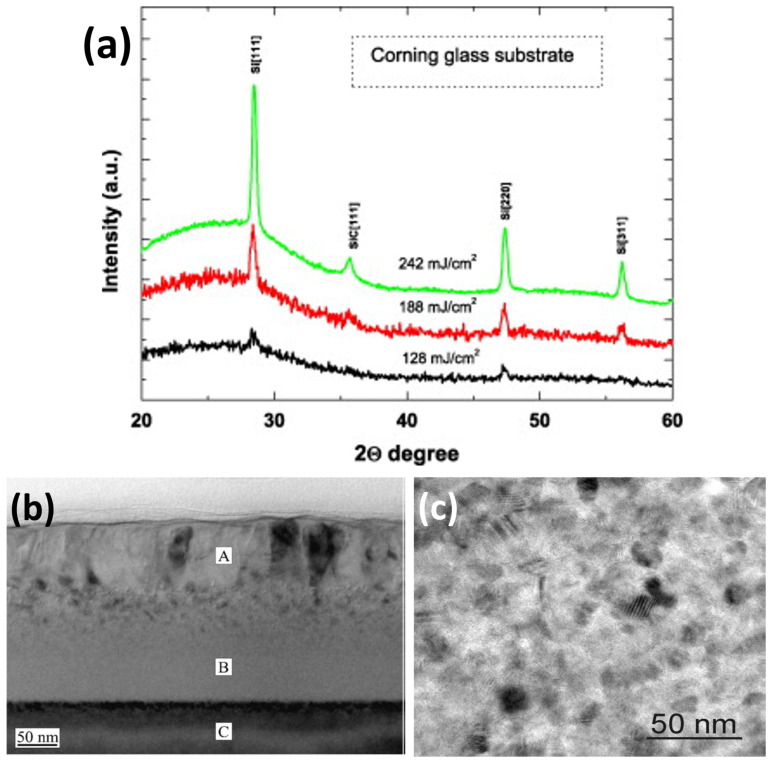
(**a**) XRD spectra of SiC samples annealed at different laser energy densities showing peaks associated with crystalline phases. Reprinted from [104], Copyright (2009), with permission from Elsevier. (**b**) TEM image indicating a polycrystalline 3C-SiC surface layer (A), a remaining amorphous layer (B), and a monocrystalline 4H-SiC substrate (C) after laser annealing treatment. The scale bar corresponds to 50 nm. Reprinted from [94], Copyright (2003), with permission from Elsevier. (**c**) TEM image of a laser-crystallized SiC film. The scale bar corresponds to 50 nm. Reprinted from [96], Copyright (2001), with permission from Elsevier.

Basa et al. [104] presented evidence of successful SiC crystallization through laser annealing. They used a KrF excimer laser emitting nanosecond UV pulses with a wavelength of 248 nm and a pulse duration of 30 ns to crystallize a SiC film previously deposited using the PECVD technique in air and at room temperature. The energy density delivered ranged from 123 to 242 mJ/cm^2^. X-ray diffraction (XRD) measurements confirmed the crystallization process. The spectra (Figure 5a) displayed peaks associated with crystalline silicon at 2*θ* = 28.3° (corresponding to the (111) reflection plane of Si), 2*θ* = 47.2° (for the (220) reflection plane of Si), and 2*θ* = 56° (for the (311) reflection plane of Si). At energy densities of 188 mJ/cm^2^ or higher, a new peak emerged at 2*θ* = 35.6°, corresponding to the (111) reflection plane of cubic silicon carbide 3C-SiC. Moreover, as the laser energy density increased, the intensities of the Si and SiC peaks also increased, providing clear evidence of improved crystallinity.

Hedler et al. [94] also utilized a nanosecond-pulsed KrF excimer laser (30 ns pulse duration) to achieve SiC structure recovery. They employed a wavelength of 248 nm to enable SiC band-to-band absorption and facilitate the crystallization of a previously amorphized SiC film by ion implantation. The experiment was conducted in air and at room temperature using up to 50,000 pulses at a repetition rate of 50 pulses/s and laser fluences ranging from 150 to 900 mJ/cm^2^. Subsequent characterization measurements confirmed the crystallization and revealed that higher laser fluences led to a deeper crystallization within the film and an oxidation effect due to the laser irradiation in the air. Additionally, transmission electron microscopy images (Figure 5b) demonstrated that an amorphous layer remained between the upper annealed polycrystalline 3C-SiC layer and the crystalline 4H-SiC substrate, indicating that no epitaxial growth occurred during the annealing process.

To achieve successful recrystallization of previously amorphized silicon carbide (SiC) through Al^+^ ion implantation, Mazzamuto et al. [105] utilized an excimer laser system with a different emitted wavelength (308 nm) in the UV range and a short pulse duration of about 160 ns. A higher irradiated fluence allowed for deeper columnar recrystallization of the SiC film after the melting phase induced by laser treatment. The effectiveness of the laser annealing process was confirmed through X-ray diffraction (XRD) measurements. The XRD signal exhibited two prominent peaks at 2*θ* = 33.5°, corresponding to the (100) orientation of 4H-SiC, and 2*θ* = 71°, corresponding to the (201) orientation of 4H-SiC. Following the annealing process, the peak at 2*θ* = 33.5° experienced a slight shift due to Al doping activation caused by laser treatment. Furthermore, electron energy loss spectroscopy (EELS) analysis clarified the potential for epitaxial regrowth of crystalline SiC. Three main regions resulting from the phenomenon of 4H-SiC epitaxial regrowth were identified: a region consisting of carbon crystallized in thin graphite layers (multi-layer graphene), a region with crystal silicon, and a region of strained 4H-SiC.

Laser annealing experiments were also conducted under various environmental conditions, including a heated sample [96], an argon (Ar) atmosphere [106,107], and under vacuum [95] to facilitate crystallization and remove oxidizing agents. Urban et al. [96] specifically employed a KrF excimer laser with a 25 ns pulse duration and a UV wavelength of 248 nm to anneal a sample placed on a heating stage at 400 °C. A single laser shot with fluences ranging from 100 to 1000 mJ/cm^2^ was applied to the material. Optical microscopy, transmission electron microscopy (TEM), and Raman spectroscopy were used for characterization, revealing the presence of a fluence threshold of 250 mJ/cm^2^. Below this threshold, no modifications occurred in the SiC samples, leaving the material in its amorphous phase. Conversely, when the SiC film was irradiated with a laser fluence higher than 250 mJ/cm^2^, the annealing process successfully crystallized the SiC film, resulting in SiC grains with diameters of 10–20 nm and cubic polycrystalline structures, as depicted by the TEM images (Figure 5c).

**Figure 6:**
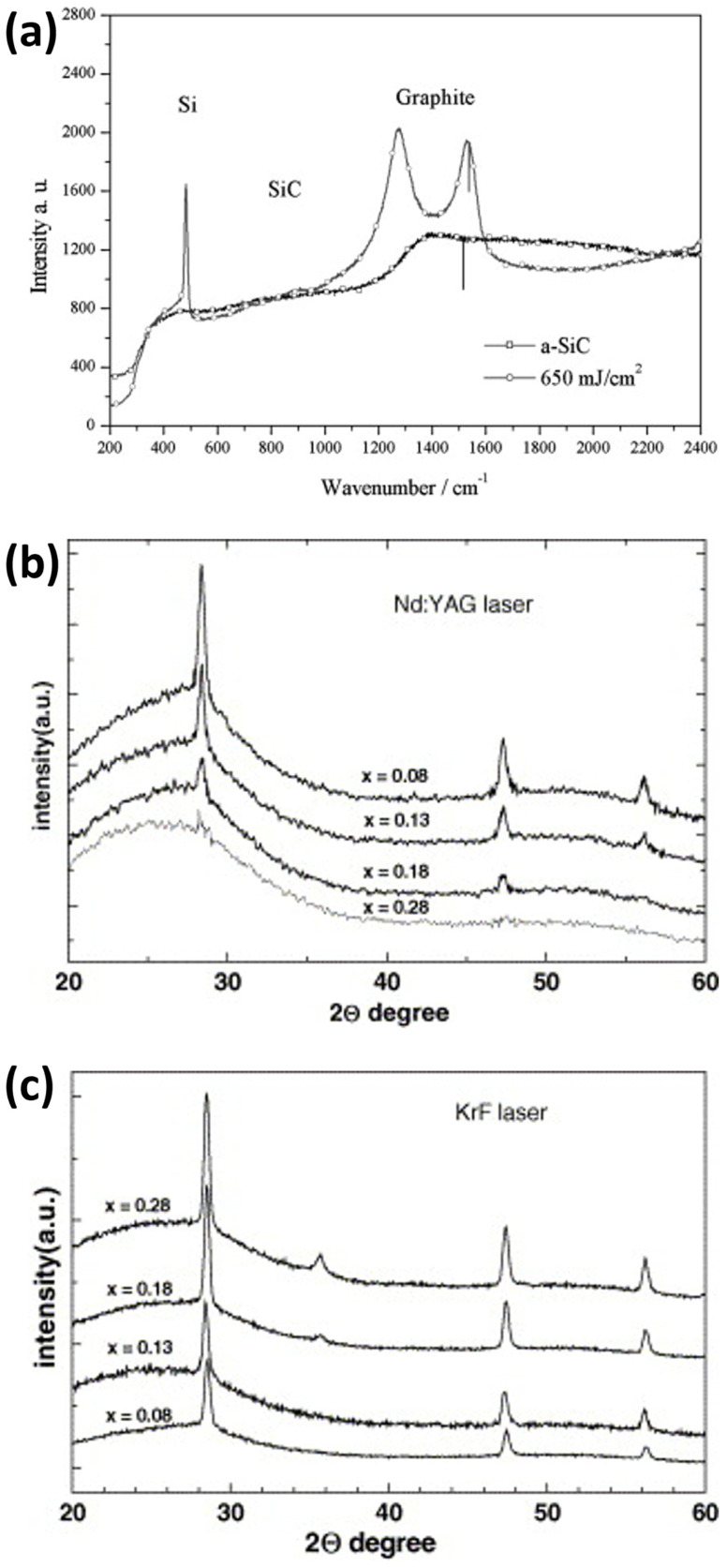
(**a**) Raman spectra of silicon carbide films before (a-SiC) and after high fluence irradiation, showing segregation into c-Si and graphite. Reprinted from [96], Copyright (2001), with permission from Elsevier. (**b**) XRD spectra of SiC films, with different carbon content x, irradiated with Nd:YAG green laser. Reprinted from [109], Copyright (2005), with permission from Elsevier. (**c**) XRD spectra of SiC films, with different carbon content x, irradiated with KrF UV laser. Reprinted from [109], Copyright (2005), with permission from Elsevier.

Fluences significantly exceeding the crystallization threshold resulted in the segregation of SiC, as shown in Figure 6a. This was indicated by the appearance of Raman peaks at 1500 cm^−1^ (graphite) and 510 cm^−1^ (crystalline Si), while the typical peaks of crystalline SiC around 790 or 960 cm^−1^ were absent. The remaining amorphous SiC near the substrate contributed to the high background signal. It is worth noting that, considering the laser parameters used by Urban et al. [96], such as a 25 ns pulse duration, solid-phase crystallization seems unlikely, suggesting that the annealing process involves a mechanism associated with a metastable liquid phase of SiC.

There have been attempts to anneal SiC films using infrared (IR) wavelength, as demonstrated in the study by Goyes et al. [108]. In their experiment, a continuous wave CO_2_ laser with a wavelength of 1060 nm and Gaussian beam distribution was employed to anneal an amorphous SiC film deposited on a silicon wafer under vacuum conditions. The irradiation time ranged from 10 to 30 min, while the laser power was set to 8 W. X-ray diffraction (XRD) measurements were taken to compare sample structures before and after laser treatment. The XRD spectra revealed that the SiC film was initially amorphous, as evidenced by the absence of peaks. However, laser annealing led to crystallization and the formation of *α*-SiC and *β*-SiC phases. Specifically, after 10 min of annealing, the *β*-SiC phase with a (200) orientation was predominant, as it is the most stable phase at high temperatures. After 30 min of annealing, *α*-SiC became the dominant crystalline phase, although *β*-SiC with a (111) orientation was still present. Silicon carbide absorption of IR light is negligible, thus crystallization of a-SIC was probably induced by heating the silicon layers underneath, which absorbed the laser radiation, and by thermal conduction of the silicon carbide thin film.

The implementation of laser radiation in the visible range has shown some promising results, allowing it to be considered a possible alternative to UV annealing, as demonstrated by several studies [93,109,110]. Baeri et al. [93] observed that annealing an amorphous SiC sample with a Q-switched ruby laser emitting at a wavelength of 694 nm (red) and fluences ranging from 100 to 1000 mJ/cm^2^, along with a 25 ns pulse duration, caused a solid-phase transformation of amorphous SiC into the crystal phase. This process involved the generation of heat through light absorption near the sample surface, followed by cooling through heat transport to the colder substrate. The amorphous SiC reached its equilibrium temperature with the liquid SiC and underwent solidification, resulting in a polycrystalline structure with an average grain size of approximately 30 nm.

Ambrosone et al. [109] investigated the effects of SiC laser annealing using a pulsed frequency Nd:YAG laser with a green wavelength (532 nm) and pulse duration and energy of 10 ns and 2.9 mJ, respectively. The results were compared with those obtained using a pulsed KrF excimer laser with a UV wavelength (248 nm) and pulse duration and energy of 30 ns and 290 mJ, respectively. Both laser annealing experiments were conducted in air at room temperature, with a 60% spatial overlap between successive pulses delivering a fluence of 242 mJ/cm^2^. The goal was to crystallize amorphous silicon films with carbon content (χ) ranging from 0.08 to 0.28. The annealed samples were subsequently characterized using XRD spectroscopy to assess how different parameters influence the structure of the final material. Figure 6b illustrates the XRD spectra of films annealed with green laser light, displaying three sharp peaks indicative of the presence of crystalline Si, while peaks associated with crystalline SiC grains were absent. Additionally, it can be observed that the amplitudes of the crystalline Si peaks decrease as the carbon content increases, resulting in a decrease in the crystalline volume fraction (evaluated by the integrated area of the peaks [111]). The film with the highest carbon content (χ = 0.28) exhibited only a small peak at 28.3°.

On the contrary, the XRD spectra in Figure 6c reveals that the peaks associated with crystalline Si become sharper and their intensities increase after UV laser annealing, indicating increasing crystalline volume fractions with higher χ values. Interestingly, crystalline SiC is also present after treatment. The spectra of films with higher χ (i.e., 0.18 and 0.28) show a peak at 35.6° corresponding to the (111) plane of 3C-SiC [109]. Similar to the peaks associated with crystalline Si, a higher carbon content (χ) results in a greater SiC crystalline volume fraction. The average size of the crystalline SiC grains is approximately 30 nm, calculated using the Debye-Sherrer formula.

This difference between the SiC absorption of green and UV lights can be theoretically explained; in fact, the energy associated with green light (532 nm) is about 2.3 eV, which is very similar to the amorphous SiC bandgap (in the range of 2–2.5 eV). This means that the energy of the green photons is just high enough to allow some electrons on the top of the valence band to be promoted to the very bottom of the conductive band. Therefore, by considering that the states in the regions of conductive and valence bands involved in this process are very few, it is possible to assert that the probability is quite low and, hence, the absorption is inefficient. Instead, the energy associated with UV photons (248 nm) is about 4.9 eV, which is much higher than the amorphous SiC bandgap; thence, there are many states involved in the promotion of electrons due to the energy released by UV photons. The absorption process is therefore very efficient in this case.

In another study by Palma et al. [110], a green laser was used for SiC annealing; however, instead of nanosecond pulses, continuous-wave radiation was emitted. The samples were irradiated in the air at room temperature using a beam with a transverse Gaussian intensity distribution delivered by an Argon laser with power densities ranging from 5 × 10^3^ to 5 × 10^6^ W/cm^2^ and a wavelength of 514.5 nm. SEM images clearly showed that different regions of the sample experienced varying degrees of SiC crystallization due to the radial intervals of the incident Gaussian energy distribution of the beam. The central region, where the temperature was higher, had polycrystalline carbon while the outer region, where the temperature was lower, had poly-Si.

After the annealing process, Raman spectroscopy was used to study the crystallization outcomes of the SiC film with a carbon content of χ = 0.3. When the sample was irradiated with a low power density (10^4^ W/cm^2^), only the Si TO phonon line at around 520 cm^−1^ was observed in the Raman spectrum [112]. Conversely, irradiating the SiC film with very high-power density (approximately 10^6^ W/cm^2^) resulted in the formation of graphite-related lines in the Raman spectrum, with broad bands at 1350 cm^−1^ and 1600 cm^−1^ and a relatively absent crystalline silicon peak.

However, using laser power density in the range of 2–6 × 10^5^ W/cm^2^, the Raman spectrum exhibited both a crystalline silicon peak at 520 cm^−1^ and broad bands at 1350 cm^−1^ and 1600 cm^−1^, characteristic of polycrystalline graphite. This indicated the simultaneous crystallization of both species, known as the phase segregation effect, highlighting the difficulty of achieving the crystallization of compound semiconductors and the stringent experimental conditions required to achieve this goal [8]. The mechanism of phase segregation in binary systems is not completely understood, but recent studies on silicon carbide demonstrate that this phenomenon can occur in solid-state binary materials that include one element that has the lowest surface energy from the liquid state of the binary system, and this element should have a larger melting temperature than the other element and the binary compound. Therefore, the presence of carbon in silicon carbide facilitates the formation of phase separation.

Lastly, it is important to note that with a slightly higher laser power density (8 × 10^5^ W/cm^2^), an amorphous SiC film with a specific carbon content of χ = 0.48 can undergo the crystallization process without the segregation effect. Consequently, the crystalline phase of SiC can be obtained after laser annealing. The Raman spectrum displayed a sharp peak at 790 cm^−1^, typical of crystalline SiC, and weaker and broader features at 900–1000 cm^−1^. This analysis confirmed the conversion of amorphous SiC into crystalline SiC. Specifically, since the hexagonal phase *α*-SiC would have exhibited a peak at 970 cm^−1^, the Raman analysis demonstrated that the produced polytype is cubic SiC (*β*-SiC). The mechanism of phase segregation in a binary system is not fully understood; however, recent studies on silicon carbide indicate that this phenomenon can occur in solid-state binary materials containing an element with the lowest surface energy from the liquid state of the binary system. Additionally, this element should have a higher melting temperature than both the other element and the binary compound. The presence of carbon in silicon carbide promotes the formation of phase separation [113]. Stoichiometric SiC (Si:C ratio around 1:1) preserves phase separation along with precise control of power density to more uniformly melt the amorphous silicon carbide layer.

A summary of the main parameters used during SiC laser annealing processes reported in the literature that attempt to crystallize amorphous silicon carbide is presented in Table 2.

Several attempts to crystallize amorphous silicon carbide through laser annealing have indicated that the most effective method involves the use of UV wavelengths. This is because UV wavelengths possess energies well above the bandgap of a-SiC. Conversely, lasers in the visible range are less effective due to inefficient absorption, as the photon energy closely aligns with the a-SiC bandgap. However, by carefully tuning process parameters, crystallization can still be achieved with visible-range lasers. Only one example of an IR laser process has been presented, but the crystallization was inefficient and linked to heating the underlying silicon bulk.

A notable distinction from silicon crystallization is that irradiating amorphous SiC alloys can yield different crystalline results. This outcome depends on the laser energy delivered and the carbon concentration in the film. The controlled formation of the SiC crystalline phase is achievable when the alloy composition is within the quasi-stoichiometric range (x ≈ 0.4–0.5). However, lower carbon content can result in phase segregation, leading to the formation of crystalline silicon and graphite.

## 5. Conclusions

This review underscores the pivotal role of laser annealing in semiconductor technology, particularly in transforming amorphous phases into localized crystalline structures, thereby offering precise control over material properties. Studies in the field highlight the numerous advantages of laser annealing over conventional furnace processes. Notably, it allows for localized annealing, preventing unintended damage to delicate regions and enabling spatial control of crystallization. Additionally, laser annealing facilitates rapid local temperature increases and operates under thermodynamic non-equilibrium conditions. Conversely, furnace annealing boasts advantages such as a simpler setup and higher scalability for industrial processes, treating multiple wafers simultaneously. However, it comes with drawbacks, including slower heating ramps and a lack of spatial selectivity in treating specific areas of the device.

In the realm of silicon—the most widely utilized semiconductor—a multitude of approaches has been explored. Various laser technologies, encompassing continuous wave, nanosecond-pulsed, and femtosecond-pulsed lasers, have been applied, producing radiation wavelengths spanning the infrared (IR), visible, and ultraviolet (UV) spectrums, yielding diverse but predominantly positive outcomes. Silicon exhibits diminished absorption of infrared (IR) light compared to visible and ultraviolet (UV) light, necessitating the use of higher power and shorter pulse durations in procedures involving IR light. Despite the challenges associated with crystallization using IR light, it remains viable, even on thicker amorphous layers, owing to its greater penetration depth. Conversely, laser annealing processes prove more efficacious in fostering crystallization with visible and UV lasers, especially on thin amorphous layers, due to silicon’s heightened absorption rate in this spectral range. A review of the literature reveals that laser annealing of silicon layers has evolved into a well-established technique for crystallization and doping activation. However, achieving the desired results demands meticulous fine-tuning. Despite this prerequisite, there is considerable potential for the successful implementation of this process, transitioning from research endeavors to an industrial context.

The growing significance of silicon carbide (SiC) in harsh conditions and optical applications has spurred research on laser annealing as a means to achieve a crystalline phase transition. However, in comparison to silicon, the exploration of laser annealing routes for SiC is relatively limited. Excimer lasers, generating nanosecond pulses in the UV range, have been the predominant technology for SiC annealing, demonstrating effective crystallization of amorphous SiC. Only a few prior experiments have delved into the use of continuous wave lasers based on non-excimer technologies or utilized wavelengths in the visible range. Further exploration of these alternative solutions, along with the adoption of new laser technologies, can provide detailed insights into achieving efficient laser annealing of SiC. This advancement would enable precise control over the transition from amorphous SiC to the desired crystalline polytype. Hence, laser annealing emerges as a compelling technique for exploring the crystallization of amorphous SiC. However, its current application is primarily confined to the research fields. The impediments to its industrial implementation stem from various limitations and complexities. These challenges encompass the need for higher-energy photons to effectively transfer energy to the material and the intricacies associated with treating alloy materials. There is a risk of inducing phase segregation with different crystalline phases of the involved atoms, necessitating fine-tuning of the laser process to avoid these phenomena.

## Figures and Tables

**Table 1:** Main parameters of silicon laser annealing processes presented in the literature.

Ref.	Laser Type	Wavelength	Pulse Duration	Beam Profile	Environment	Energy/Power Density *
[35]	Yitterbium doped fiber	IR (1064 nm)	200 ns	/	ambient conditions	/
[36]	Ti-Sapphire	IR (800 nm)	50–125 fs	/	/	38–63 mJ/cm^2^
[38]	Ti-Sapphire	IR (800 nm)	/	/	/	160–305 mJ/cm^2^
[37]	Yb:KGW	IR(800 nm)	40–200 fs	Gaussian	/	49–69 mJ/cm^2^
[39]	Yb:KGW	IR(1026 nm)	230 fs	Gaussian	/	150 mJ/cm^2^
[45]	Ti-Sapphire	IR (800 nm)	50 fs	/	/	/
[34]	Ti-Sapphire	UV (400 nm)	50 fs	/	/	20–30 mJ/cm^2^
[33]	Nd:YVO_4_	UV (355 nm)	12–15 ns	Gaussian	/	240 mJ/cm^2^
[33]	Nd:YVO_4_	Green (532 nm)	12–15 ns	Gaussian	/	478 mJ/cm^2^
[53]	Nd:YVO_4_	Green (532 nm)	CW	Gaussian in short axis, top-hat in long axis	/	/
[44]	Solid-state diode	Blue (440 nm)	CW	Elliptically	/	4.61 W
[54]	GaN-based diode	Blue (450 nm)	CW	Gaussian	room T, N_2_ atmosphere	/
[54]	Nd:YAG	Green (532 nm)	30 ns	rectangular	room T, N_2_ atmosphere	153 mJ/cm^2^

* Since the CW (continuous wave) lasers have no pulses, a power density is defined instead of an energy density.

**Table 2:** Main parameters of silicon carbide laser annealing processes presented in the literature.

Ref.	Laser Type	Wavelength	Pulse Duration	Beam Profile	Environment	Energy/Power Density *
[104]	KrF excimer	UV (248 nm)	Nanosecond (30 ns)	/	Room T, air	123–242 mJ/cm^2^
[94]	KrF excimer	UV (248 nm)	Nanosecond (30 ns)	/	Room T, air	150–900 mJ/cm^2^
[105]	LASSE excimer	UV (308 nm)	Nanosecond (160 ns)	/	/	3200 mJ/cm^2^
[96]	KrF excimer	UV (248 nm)	Nanosecond (25 ns)	/	T = 400 °C, air	100–1000 mJ/cm^2^
[106]	KrF excimer	UV (248 nm)	Nanosecond (20 ns)	/	Ar atmosphere	200 mJ/cm^2^
[95]	XeCl excimer	UV (308 nm)	/	/	Room T, vacuum	/
[82]	XeCl excimer	UV (308 nm)	Nanosecond (160 ns)	/	/	1000–2800 mJ/cm^2^
[83]	XeCl excimer	UV (308 nm)	Nanosecond (30 ns)	/	/	500–600 mJ/cm^2^
[107]	Nd:YAG	UV (355 nm)	Nanosecond (10 ns)	Gaussian	Ar atmosphere	100–1200 J/cm^2^
[109]	KrF excimer	UV (248 nm)	Nanosecond (30 ns)	/	Room T, air	242 mJ/cm^2^
[109]	Nd:YAG	Green (532 nm)	Nanosecond (10 ns)	/	Room T, air	242 mJ/cm^2^
[93]	q-switched Ruby	Red (694 nm)	Nanosecond (25 ns)	/	/	100–1000 mJ/cm^2^
[110]	Argon laser	Green (514 nm)	Continuous wave	Gaussian	Room T, air	8 × 10^5^ W/cm^2^
[5]	Nd:YLF	Green (527 nm)	Nanosecond (200 ns)	/	N_2_ atmosphere	1170–2500 J/cm^2^
[108]	CO_2_	DIR (1060 nm)	Continuous wave	Gaussian	Vacuum	5.7 W/cm^2^

* Since the CW (continuous wave) lasers have no pulses, a power density is defined instead of an energy density.

## Data Availability

Not applicable.
